# Effect of physio-chemical seed treatments on opium poppy downy mildews caused by *Peronospora meconopsidis* and *P*. *somniferi*

**DOI:** 10.1371/journal.pone.0230801

**Published:** 2020-04-10

**Authors:** Tamilarasan Thangavel, Jason Scott, Suzanne Jones, Ramya Gugalothu, Calum Wilson

**Affiliations:** 1 Research Laboratories, Tasmanian Institute of Agriculture (TIA), University of Tasmania (UTAS), New Town Australia; 2 TIA, UTAS, Burnie, Tasmania, Australia; Universita degli Studi di Pisa, ITALY

## Abstract

Downy mildew of opium poppy is the single biggest disease constraint afflicting the Australian poppy industry. Within the pathosystem, the transmission of infections via infested seed is of major concern. Both downy mildew pathogens of poppy; *Peronospora meconopsidis* and *P*. *somniferi*, are known contaminants of commercial seed stocks. Using seed naturally infested with these pathogens, the effect of physio-chemical seed treatments on seedling health and disease transmission were evaluated. Individual seed treatments were tested to determine optimal treatment parameters for each; including incubation time, temperature and treatment concentration. Optimised physiochemical treatments were then compared. The most effective treatment methods were seed washes in acidified electrolytic water (400 ppm hypochlorous acid for 5 min) and hypochlorite solution (2% NaOCI for 5 min). In seed to seedling transmission assays, these two treatments reduced transmission of *P*. *somniferi* by 88.8% and 74.61%, and *P*. *meconopsidis* by 93.3% and 100%, respectively. These methods are recommended for seed treatment of commercial opium poppy seed to assist in the control of the downy mildew diseases.

## Introduction

Opium poppy (*Papaver somniferum*) is a highly valuable annual crop grown primarily for its opiate alkaloids. Around 40 alkaloids, including morphine, thebaine, codeine and noscapine, can be isolated from poppy. These alkaloids are used primarily by pharmaceutical industries in the synthesis of opiate-based medicinal products [1, 2]. Australia is the world’s largest producer of licit opiate alkaloids, contributing over half the world’s production [3, 4]. Within Australia, the majority of poppy production occurs within the island state of Tasmania [2].

Downy mildew is one of the most destructive diseases of poppy worldwide and causes substantial economic losses to the Australian poppy industry [5, 6]. Two pathogen species, *Peronospora meconopsidis* and *P*. *somniferi*, can cause downy mildew, with each associated with a distinct set of symptoms. Infections by *P*. *meconopsidis* are characterized by localised angular lesions on the adaxial surface of leaves and sparse sporulation on the abaxial surface [7, 8]. Prior to 2014, this was the only form of the disease known in Australia. However since 2014, systemic plant infections resulting in stunted plant growth, leaf deformation and heavy sporulation on the abaxial leaf surfaces have been observed. These symptoms are associated with infections by *P*. *somniferi* [6].

Understanding the drivers for pathogen spread is a large component of developing management for plant diseases. In poppy, transmission of downy mildew pathogens from seed-borne inocula to seedlings has been demonstrated [5, 9]. While transmission rates are generally low, seed transmission is an important mechanism for initiating epidemics and creating in field foci of inocula for subsequent secondary spread within the crop [8, 10]. As such, minimising seed transmission is seen by the poppy industry as an important component of managing the disease.

A large number of seed treatments, that are either physical, chemical, or biological in nature have been considered for management of seed-borne pathogens, including downy mildews [11]. Chemical fungicides are already a major component of the disease management strategy of the poppy industry, particularly for the control of secondary spread of downy mildew. Thus, alternative seed treatments that do not further increase reliance on fungicides are perceived as beneficial by the industry. Many alternatives to chemical fungicides can be used for such treatments. These include treating seed with oxidised chlorine compounds such as hypochlorite [12–15] or electrolyzed water solutions [16–20] and incubating seed at elevated treatments in hot water [21–24] or with aerated steam [25–28]. However, such treatments require evaluation and optimisation for individual pathosystems, as treatment protocols involve balancing the effects on pathogen inoculum control with maintaining physio-chemical seed health and vigour [26, 28–32].

This study aimed to evaluate the potential of select non-fungicide seed treatments for reducing transmission of both *Peronospora* spp. from seed to developing poppy seedlings. That such treatments, ensured optimal seed viability and performance was also a key consideration.

## Materials and methods

### Poppy seed

Poppy commercial seed lines SL21 and SL22, previously shown to contain high levels of both *P*. *somniferi* and *P*. *meconopsidis* inocula [5], were supplied by the Australia poppy industry for this study. Seed was stored at ambient room temperature (18–22°C) in the dark before use. In all experiments 10 g of seeds (equating to ~10,000 seeds) were used per seed treatment.

### Seed treatments

Four seed treatment methods were evaluated in this study. These can be broadly grouped as either chlorine- (hypochlorite or electrolyzed water) or heat- (hot water or aerated steam) based. To optimise the protocols, each treatment was first evaluated individually. This was followed by two experiments comparing the efficiency of select individual seed treatments (Table 1). In all experiments, untreated controls were included; for studies involving electrolyzed water, hot water and hypochlorite, control seeds were soaked at ambient room temperature (18–22°C) in sterile water for an equivalent time-period. The controls for steam treatment studies were seed exposed to ambient room air (18–22°C). Following treatment, all replicates from each treatment were assessed for seed germination, microbial contamination, and seedling emergence. In all experiments, seed lot SL 21 was used unless otherwise mentioned. All treatments in all experiments were replicated in triplicate.

**Table 1:** Outline of seed treatment experiments and treatment parameters conducted in this study.

Experiment code	Treatment type	Incubation time (min)	Chlorine concentration^a^	Incubation Temperature (°C)
BT1	Hypochlorite solution	0, 5, 10, 15 & 20	0, 1, 2, 3 & 4	RT^b^
BT2		5	0, 1, 2, 3 & 4	RT and 5
EW1	Hypochlorous acid	0, 5, 10, 15, 30 & 60	0, 50, 100, 200 & 400	RT and 0, 5, 10, 15, 30 & 60
EW2		0, 5, 10, 15 & 20	0, 200 & 400	RT and 0, 5, 10, 15 & 20
HW1	Hot water	5, 15, 20 & 30	-	RT, 30, 40, 50& 60
HW2		15	-	RT, 30, 40, 50 & 60
HW3		15	-	RT, 40, 50, 60 & 70
ST1	Aerated steam	15, 30 & 60	-	RT, 40, 50 & 60
ST2		10, 20, 30 & 40	-	RT, 40, 50 & 60
ST3		15	-	RT, 40, 50, 55, 60, 65 & 70
Protocol comparison 1	Untreated	0	-	RT
	Hypochlorite solution	5	2	RT
	Hypochlorous acid	15	200	RT
		15	400	RT
	Hot water	15	-	40
		15	-	50
		15	-	60
		15	-	70
	Aerated steam	15	-	50
		15	-	60
	Hypochlorous acid + Hot water	15 (HOCI), 15 (Hot water)	200	RT, 40 (Hot water)
		15 (HOCI), 15 (Hot water)	200	RT, 60 (Hot water)
	Hypochlorous acid + Aerated steam	15 (HOCI), 15 (Aerated steam)	200	RT, 60 (Aerated steam)
Protocol comparison 2	Untreated	0	-	RT
	Hypochlorite solution	5	1	RT
		5	2	RT
		5	4	RT
	Hypochlorous acid	15	100	RT
		15	200	RT
		15	400	RT
	Hot water	15	-	30
		15	-	40
		15	-	50
		15	-	60
	Aerated steam	15	-	40
		15	-	50
		15	-	60
		15	-	70
	Hypochlorite solution + Hypochlorous acid	5 (NaOCl), 15 (HOCl)	2 & 400	RT, RT
	Hypochlorite solution + Hot water	5 (NaOCl), 15 (HW)	2 & 400	RT, 40 (Hot water)
	Hypochlorite solution + Aerated steam	5 (NaOCl), 15 (Aerated steam)	2	RT, 60 (Aerated steam)
	Hypochlorous acid + Aerated steam	5 (NaOCl), 15 (Aerated steam)	200	RT, 60(Aerated steam)
	Hot water + Hypochlorous acid	5 (NaOCl), 15 (HOCl)	200	40 (Hot water), RT
	Hot water + Aerated steam	15 (Hot water), 15 (Aerated steam)	-	40 (Hot water), 60 (Aerated steam)

^a^ Concentration of active chlorine in solution measured as a percentage (%; hypochlorite treatments) or parts per million (ppm; hypochlorous acid treatments).

^b^ RT, ambient room temperature (18–22°C).

### Hypochlorite treatment

The optimal condition for hypochlorite treatment of seed was evaluated in two rounds of testing (BT1 and BT2; Table 1). A commercial bleach solution with sodium hypochlorite (42 g/L), 4% available chlorine (White King Bleach Standard, Victoria, Australia) was used in both experiments. In BT1, 10 g of seed were placed in a 100 ml conical flask and 50 ml of NaOCl solution added. Concentrations of 0 (control), 1, 2, 3 and 4% available chlorine were used, with incubation times of 0, 5, 10, 15 and 20 min. Treated seed was then rinsed three times in sterile deionised water, drained on sterile paper towel, and air-dried at ambient room temperature (18–22°C) in a laminar flow chamber for 24 h. In BT2, the same NaOCl concentrations were repeated, but for only a single incubation time (5 min). Treated seeds were rinsed, drained and air dried as previously described.

### Electrolysis water treatment

Electrolysis water solution was obtained as electrolyzed saline water (generated using the Envirolyte^®^ EL-400, Envirolyte Industries International OÜ, Tallinn, Estonia) from Prof Roger Stanley (Centre for Food Safety, Newnham campus, University of Tasmania). The chlorine concentration of the electrolysis water was determined by colorimetric analysis using a commercial kit (Compact ClO_2_+ meter, Palintest, Australia). In both experiments, 10 g of seed was placed in a 100 ml conical flask and then 50 ml of electrolysis water solution was added. In EW1, seeds were treated in 0 (control), 50, 100, 200 and 400 ppm ClO_2_ (pH = 6.7) for 0, 10, 15, 30 and 60 min. In EW2, seeds were treated at 0, 200 and 400 ppm (pH = 6.7) for 0, 5, 10, 15 and 20 min. Following treatment, seeds were drained onto a sterile paper towel and air dried at ambient room temperature (18–22°C), as previously described.

### Hot water treatment

In three experiments (HW1, HW2 and HW3), 10 g of seed was placed into a 100 ml conical flask that contained 50 ml of sterile deionised water at ambient room temperature (18–22°C). The flasks were then incubated in a hot water bath (Ratek Instruments Pty Ltd, Australia) set at the various test temperatures. A thermometer (76mm N_2_ filled immersion Zeal, England) was placed inside the flask to monitor temperature. The duration of individual treatments commenced from the time the water attained the desired temperature. In HW1, seeds were incubated at 30°C, 40°C, 50°C and 60°C for durations of 0, 5, 15, 20 and 30 min. In HW2, seeds were incubated at 40°C, 50°C, 60°C and 70°C for 15 min only. In HW3, seeds incubated at 30°C, 40°C, 50°C and 60°C for 0, 5, 15 and 30 min. Following treatment, seeds were drained and air dried at ambient room temperature (18–22°C), as previously described.

### Aerated steam treatment

In three experiments (ST1, ST2 and ST3), 10 g of seeds were spread evenly across the base of open Petri plates (60 × 15 mm) and placed in a steamer oven (Royston steamer DS745, Hospitality World Direct, Australia) at ambient room temperature (18–22°C). Target temperatures were set and monitored using a thermometer within the chamber. The duration of individual treatments commenced from the time the internal chamber attained the desired temperature. In ST1, seeds were treated at 40°C, 50°C and 60°C for 0, 15, 30 and 60 min. In ST2, seeds were treated at 40°C, 50°C and 60°C for 0, 10, 20, 30 and 40 min. In ST3, seeds were treated at 40°C, 50°C, 55°C, 60°C, 65°C and 70°C for 0 and 15 min. Treated seeds were then air dried at room ambient room temperature(18–22°C), as previously described.

### Treatment protocol comparison

Selected optimised treatments of the four seed disinfestation methods and selected combinations of treatments were directly compared in two protocol comparisons experiments (PC1 and PC2). Each experiment used 10 g of seed; PC1 used seed lot SL21 and PC2 used seed lot SL22. Both experiments tested a set of individual treatment types as well as compound treatments of varying conditions (Table 1).

A completely randomized block design was adopted for the seed emergence and seed-disease transmission experiments. In all experiments, three biological replicates were conducted for each treatment.

### Seed health assessment

#### Seed germination and contamination rate

Germination capacity and microbial contamination of treated seed was tested in all experiments. Twenty-five seeds were sampled following each seed treatment and were aseptically placed onto four 2% water agar plates (*n* = 100) and incubated at 20°C with a 16 h photoperiod under cool white fluorescent lamps (65 μmol/m^**2**^s^**1**^). After 10 days, seeds were assessed for germination and presence of any fungal or bacterial contaminants on the medium. For germination, only seeds that produced true leaves were counted. For contamination, both non-germinated and germinated seeds with fungal or bacterial colonies present were considered contaminated.

#### Seedling emergence

From each experiment, 100 seeds (*n* = 100) were sampled and sown in plastic trays (54 cm x 28 cm) filled with pasteurised potting mix containing sand, peat, and composted pine bark (10:10:80, pH 6.0) premixed with Osmocote 16–3.5–10 NPK resin coated fertilizer (Scotts Australia Pty Ltd.) to a depth of 0.5 cm. Glasshouse temperatures were maintained at 15–18°C by day with relative humidity at 55–60% and 10–14°C at night with 70–75% relative humidity. Trays were hand watered daily to maintain soil moisture. Trays were arranged in a randomized complete block design. Seedling emergence was determined three weeks after sowing.

#### Seed-disease transmission and disease incidence

To test the efficacy of individual treatments on seedborne-pathogen transmission to seedlings, and on subsequent visual disease incidence, glasshouse trials were conducted using the seeds treated in experiments BT1, EW2, HW1, ST1, PC1 and PC2. For each treatment, 3 g of seeds were sown on seed raising trays (60 × 40 × 12 cm) that were filled with a potting mix. In each tray, 0.5 g seeds were sown and covered with 0.5 cm layer of potting mix. In total six replicate trays were sown for each treatment. Experiments were conducted within a glasshouse environment with temperatures maintained as above. Hand watering occurred twice daily avoiding watering directly onto leaves.

Above ground foliage from three-week-old seedlings from each tray were harvested and tested for the presence of *P*. *somniferi* and *P*. *meconopsidis* using a pathogen species-specific PCR assay [5]. For each treatment, 15 groups of 100 seedlings were sampled from each replicate. Grouped seedlings were ground using a mortar pestle and 200 **μ**l of homogenised plant tissue used for DNA extraction according to manufacturer’s protocol using a Power Plant Pro DNA isolation kit(Mo BIO laboratories, Australia). Samples were then tested for each pathogen using the species-specific PCR assay [5]. Pathogen incidence was subsequently estimated using the group testing methodology of Gibbs and Gower [33].

After PCR testing, plants were visually assessed for disease. Individual trays were sealed with a plastic bag to enhance sporulation, raising the relative humidity to 100% for 24 hrs. After three days, 100 plants (*n = 100*) were assessed for disease based on the following symptoms: for *P*. *meconopsidis* infection symptoms were localized polyangular lesions surrounded by chlorosis and symptoms; for *P*. *somniferi* infections symptoms were localized or systemic infection, stunted plants or leaves and strongly distorted stems.

### Data analysis

Estimates of seed germination, contamination, emergence, and the incidence of *Peronospora* spp. in seedlings were analysed individually for treatment differences in the all experiments. Observations were fit using logistic regression modelling in the R statistical language framework v. 3.4 [34]. For *in vitro* seed testing, the counts of germinated or contaminated seeds were regressed with the total number of seedlings per replicate as the negative case. For seedling germination, the count of germinated seedlings at three weeks post-sowing was regressed with total number of seeds sown per replicate. Incidence of *P*. *somniferi* and *P*. *meconopsidis* in germinated seedlings was assessed by analysing the number of positive and negative qPCR reactions (*n* = 15) obtained per replicate. Post hoc comparisons were undertaken using least-squares means estimates incorporating the Tukey correction for multiple pairwise comparisons using the lsmeans package in R [35].

## Results

### Hypochlorite treatment

Following hypochlorite treatment, seed germination rates were typically improved relative to untreated seed (Table 2). Across both experiments, the highest rate of germination achieved was for seed treated with 2% hypochlorite solutions for 5 min (95 and 94% for experiments BT1 and BT2, respectively). Increasing either treatment duration or hypochlorite concentrate resulted in decreased seed germination rates. The highest observed reduction in seed contamination occurred when treatment duration was increased to 15 or 20 min (Table 2). Contamination rate was significantly reduced relative to untreated seed when concentrations were greater than 1%, irrespective of treatment duration, and when treated with a 1% solution for 15 min or more in BT1 (Table 2). However, in BT1 differences between individual treatment combinations were not significant at concentrations greater than 1%. In BT2, contamination rate was lowest when treated with a 4% solution, which was significantly less than all other concentrations. Seedling emergence was highest in seed treated with 2% hypochlorite for 5 min in BT1, but significantly decreased with increasing hypochlorite concentration in BT2 (Table 2).

**Table 2:** The effect of hypochlorite solution treated at various concentration and time on the opium poppy seeds. The mean proportion of germination, emergence, contamination and disease incidences were estimated using logistic regression model. Significant differences were grouped using HSD mean separation.

Hypochlorite solution (%)	Time (min)	Germination (%) ^a^	Contamination (%) ^b^	Emergence (%) ^b^	Systemic downy mildew incidence ^c^	Localised downy mildew incidence ^c^	*P*. *somniferi* transmission/incidence in seedlings ^d^	*P*. *meconopsidis* transmission/incidence in seedlings ^d^
0	0	52.7 a	17.7 e	47.7 ab	2.0	0.3	5.3 c (2.9)	1.0 (0.47)
1	5	62.4 ab	10.7 cde	61.4 bcd	0.0	0.0	3.3 bc (1.73)	0.3 (0.16)
1	10	80.0 cdef	6.7 abcd	56.0 abcd	0.3	0.0	2.3 abc (1.14)	0.3 (0.16)
1	15	80.0 cdef	7.7 bcd	52.0 abc	0.0	0.0	2.0 abc (0.97)	0.0 (0.00)
1	20	86.7 efgh	3.0 ab	60.0 bcd	0.3	0.0	0.3 ab (0.16)	0.3 (0.16)
2	5	96.0 i	6.7 abcd	74.7 e	0.0	0.0	0.3 ab (0.16)	0.3 (0.16)
2	10	84.0 defg	2.4 ab	63.4 cde	0.0	0.0	0.3 ab (0.16)	0.0 (0.00)
2	15	94.7 hi	13.7 de	58.7 abcd	0.0	0.0	0.3 ab (0.16)	0.0 (0.00)
2	20	91.4 ghi	5.7 abcd	64.0 cde	0.0	0.0	0.3 ab (0.16)	0.0 (0.00)
3	5	88.0 fgh	4.7 abc	52.0 abc	0.0	0.0	0.3 ab (0.16)	0.0 (0.00)
3	10	72.0 bc	3.4 ab	65.4 cde	0.0	0.0	0.0 a (0.00)	0.0 (0.00)
3	15	86.7 efgh	1.4 a	66.7 de	0.0	0.0	0.3 ab (0.16)	0.0 (0.00)
3	20	74.7 bcd	2.7 ab	54.7 abcd	0.0	0.0	0.3 ab (0.16)	0.0 (0.00)
4	5	81.4 cdef	6.1 abcd	49.4 ab	0.0	0.0	0.0 a (0.00)	0.0 (0.00)
4	10	73.4 bcd	4.0 abc	54.0 abcd	0.0	0.0	0.0 a (0.00)	0.0 (0.00)
4	15	77.4 cde	2.0 ab	45.7 a	0.0	0.0	0.0 a (0.00)	0.0 (0.00)
4	20	84.0 defg	5.4 abc	48.0 ab	0.0	0.0	0.0 a (0.00)	0.0 (0.00)
* Probability*^***d***^	* *	*<0*.*001*	*<0*.*001*	*<0*.*001*	*0*.*49*	*1*.*00*	*<0*.*001*	*0*.*60*
0	5	71.9 a	0.2 d	69.5 c	-	-	-	-
1	5	82.0 a	0.1 c	65.0 bc	-	-	-	-
2	5	93.7 c	0.0 bc	0.8 d	-	-	-	-
3	5	85.0 a	0.0 b	57.4 ab	-	-	-	-
4	5	63.4 a	0.0 a	53.7 a	-	-	-	-
*Probability*^***d***^		*<0*.*001*	*<0*.*001*	*<0*.*001*				

^a^ Values were obtained from 100 seeds plated onto water agar.

^b^ Values were obtained from 100 plants in three-week-old seedlings for both emergence and visual disease symptoms.

^c^ Results were obtained from 15 subsamples of 10 three-week-old seedlings from individual seed treatment by PCR. In PCR, weak positive amplifications were also considered as a positive detection [5]. Values in parentheses indicate the estimated pathogen transmission incidence (%) of based on the testing procedure of Gibbs and Gower [33]

^d^ Treatment differences were analysed via logistic regression. Germination, contamination, emergence and symptom incidences were analysed using number of positive seeds/seedlings out of the total number assessed. Incidence of *P*. *somniferi* and *P*. *meconopsidis* were analysed using the number of positive PCR reactions out of the total number of tests conducted. Post hoc comparisons were undertaken using least square means estimates with a Tukey correction for multiple pairwise comparisons. Treatment estimates sharing a lowercase letter within an experiment are not significantly different from each other (*P* ≥ 0.05).

No significant difference was found in the mean visual disease incidence of localised or systemic downy mildew symptoms with incidence low (≤2%) even in untreated seed (Table 2). However, it is worth noting that localised lesions were not observed in any seedling treated with hypochlorite prior to planting and systemic symptoms were only observed sporadically. Similarly, no significant differences were found in the incidence of *P*. *meconopsidis* in germinated seedlings between treatments, however incidence was only estimated to be 1% in untreated seedlings. Estimated incidence of *P*. *somniferi* in untreated seedlings was 5.4% and was reduced to 2% or less by treatment with 2% hypochlorite or higher. Treatment with 4% hypochlorite reduced *P*. *somniferi* incidence below detection limits regardless of treatment duration.

### Electrolysis water treatment

Seed treatment with 400 ppm electrolysis water significantly increased seed germination rate compared to the untreated controls regardless of treatment duration (Table 3). At lower concentrations, germination response varied with treatment duration. However, in EW1 optimal germination was achieved with 15 min treatment for 50, 100 and 200 ppm concentrations and was significantly greater than nontreated seed. In EW2, treatment with electrolysis water significantly increased germination for all concentrations and durations, with maximal germination achieved with treatment for 15 min with 400 ppm. Similarly, microbial contamination was significantly reduced by treatments at 400 ppm electrolysis water at all tested durations across both experiments. Seedling emergence was significantly greater than nontreated when treated with either 200 or 400 pm for 15 min or longer in EW1 and 5 min or longer in EW2.

**Table 3:** The effect of electrolysis water treated at various concentration and time on the opium poppy seed health and controlling poppy downy mildew.

Experiment	Hypochlorous acid (ppm)	Time (min)	Germination (%) ^a^	Contamination (%) ^a^	Emergence (%) ^b^	Systemic symptom incidence (%) ^c^	Localised symptom incidence (%) ^c^	*P*. *somniferi* incidence in seedlings ^d^	*P*. *meconopsidis* incidence in seedlings ^d^
EW1	0	0	55.3 ab	25.3 c	46.7 a	-	-	-	-
	50	5	72.3 cde	20.7 bc	55.7 abc	-	-	-	-
	50	10	59.3 abc	16.3 b	45.3 ab	-	-	-	-
	50	15	69.7 cd	20.3 bc	49.7 abc	-	-	-	-
	50	30	62.0 bc	17.3 bc	50.3 abc	-	-	-	-
	50	60	64.0 bc	16.7 b	44.7 ab	-	-	-	-
	100	5	65.3 bc	16.0b	46.7 abc	-	-	-	-
	100	10	84.0 efg	17.7 bc	45.7 ab	-	-	-	-
	100	15	92.3 gh	18.0 bc	45.7 ab	-	-	-	-
	100	30	90.0 fgh	15.0 b	57.7 abcd	-	-	-	-
	100	60	81.7 def	19.7 bc	48.7 abc	-	-	-	-
	200	5	59.7 abc	16.3 b	48.0 abc	-	-	-	-
	200	10	81.3 def	15.7 b	49.7 abc	-	-	-	-
	200	15	93.0 gh	13.0 b	60.7 cd	-	-	-	-
	200	30	87.3 fg	13.7 b	69.7 d	-	-	-	-
	200	60	47.0 a	17.7 bc	58.0 bcd	-	-	-	-
	400	5	85.0 fg	11.3 b	56.7 abcd	-	-	-	-
	400	10	96.3 h	10.3 b	51.3 abc	-	-	-	-
	400	15	92.7 gh	1.7 a	59.0 bcd	-	-	-	-
	400	30	90.7 fgh	1.7 a	58.3 bcd	-	-	-	-
	400	60	72.7 cde	0.3 a	59.0 bcd	-	-	-	-
*Probability*^***d***^	* *	* *	*<0*.*001*	*<0*.*001*	*<0*.*001*				
EW2	0	0	45.7 a	17.4 c	41.0 a	6.4 b	2.4	3.6 c (6.3)	2.0 b (4.0)
	200	5	75.0 bc	15.4 bc	65.0 bc	0.0 a	0.0	1.3 bc (2.6)	0.0 a (0.0)
	200	10	84.0 cd	12.7 bc	75.7 cd	0.0 a	0.0	1.6 bc (3.3)	0.7 ab (1.6)
	200	15	71.0 b	11.7 bc	69.7 bc	0.0 a	0.0	1.6 bc (3.3)	0.0 a (0.0)
	200	20	74.7 bc	8.7 b	63.4 b	2.4 ab	0.0	1.3 bc (2.6)	0.1 a (0.3)
	400	5	71.7 b	2.0 a	68.0 bc	0.0 a	0.0	0.4 ab (1.0)	0.1 a (0.3)
	400	10	85.4 d	1.7 a	73.0 bc	0.0 a	0.0	0.0 a (0.0)	0.1 a (0.3)
	400	15	93.4 e	0.0a	85.0 d	0.0 a	0.0	0.0 a (0.0)	0.0 a (0.0)
	400	20	74.0 bc	0.0 a	64.7 bc	0.0 a	0.0	0.0 a (0.00)	0.0 a (0.0)
*Probability*^***d***^	* *	* *	*<0*.*001*	*<0*.*001*	*<0*.*001*	*<0*.*001*	*0*.*16*	*<0*.*001*	*<0*.*001*

^a^ Values were obtained from 100 seeds plated onto water agar.

^b^ Values were obtained from 100 plants in three-week-old seedlings for both emergence and visual disease symptoms.

^c^ Results were obtained from 15 subsamples of 10 four-week-old seedlings from individual seed treatment by PCR. In PCR, weak positive amplifications were also considered as a positive detection [5]. Values in parentheses indicate the estimated pathogen transmission incidence (%) of based on the testing procedure of Gibbs and Gower [33]

^d^ Treatment differences were analysed via logistic regression. Germination, contamination, emergence and symptom incidences were analysed using number of positive seeds/seedlings out of the total number assessed. Incidence of *P*. *somniferi* and *P*. *meconopsidis* were analysed using the number of positive PCR reactions out of the total number of tests conducted. Post hoc comparisons were undertaken using least square means estimates with a Tukey correction for multiple pairwise comparisons. Treatment estimates sharing a lowercase letter within an experiment are not significantly different from each other (*P* ≥ 0.05).

The estimated visual disease incidence of symptoms of systemic downy mildew in seedlings grown from seed treated with all hypochlorous acid rates and durations, except 200 ppm for 20 min, was below detection limits and significantly less than the nontreated seed (Table 3). Localised downy mildew symptoms were only detected in nontreated seedlings. Incidence of *P*. *somniferi* and *P*. *meconopsidis* within seedlings was significantly reduced by treatment with 400 ppm electrolysis water at all durations. Additionally, *P*. *meconopsidis* incidence in seedlings treated with 200 ppm for 15 and 20 min was also significantly reduced compared to nontreated seedlings (Table 3).

### Hot water treatment

Treatment of seed with hot water at 40°C, regardless of duration, or 50°C for 20 min or less was associated with significant increases in germination rate, relative to the control, across all three experiments (Table 4). However, contamination rate was not reduced unless treated at 40°C for 30 min, or at higher temperatures. Both seed germination and contamination were significantly reduced by treatment at 60°C or higher. Seedling emergence was not significantly different from the control at 30°C and was significantly reduced by treatment at 60°C or higher. Incidence of downy mildew was ≤1.2% for both systemic and localised symptoms with no significant treatment effects detected (Table 4). However, incidence of both symptom types was below detection limits when treated with 50 or 60°C regardless of treatment duration. Similarly, incidence of *P*. *meconopsidis* infection was low and no significant treatment effects were observed. Conversely, incidence of *P*. *somniferi* infection was significantly reduced by treatment at 50°C for 15 or 30 min and at 60°C for 15 min or longer.

**Table 4:** The effect of hot water treatment at various temperature and time on the opium poppy seed health and controlling poppy downy mildew.

Experiment code	Temp (°C)	Time (min)	Germination (%) ^a^	Contamination (%) ^a^	Emergence (%) ^b^	Systemic symptom incidence (%) ^b^	Localised symptom incidence (%) ^b^	*P*. *somniferi* incidence in seedlings ^c^	*P*. *meconopsidis* incidence in seedlings ^c^
	RT^e^	0	48.0 b	16.1 d	44.7 cd	0.3	0.3	2.3 def (4.3)	0.2 (0.5)
HW1	30	5	64.0 d	15.5 d	46.2 cd	1.2	0.2	3.0 f (5.5)	0.3 (0.6)
	30	15	64.0 d	14.5 d	46.0 cd	1.2	0.2	2.0 def (3.8)	0.5 (1.1)
	30	20	50.0 bc	15.1 d	40.2 c	1.2	0.2	1.6 cdef (3.1)	0.2 (0.5)
	30	30	50.0 bc	10.0 cd	40.0 c	1.2	0.2	2.0 def (4.0)	0.4 (0.8)
	40	5	75.0 e	13.7 d	58.0 ef	0.2	0.2	2.6 ef (4.8)	0.5 (1.1)
	40	15	75.0 e	14.0 d	57.9 ef	0.2	0.2	2.9 f (5.3)	0.1 (0.1)
	40	20	61.0 d	15.1 d	52.0 de	0.2	0.2	2.7 f (5.0)	0.3 (0. 7)
	40	30	61.0 d	4.1 b	63.2 fg	0.2	0.2	1.8 cdef (3.5)	0.1 (0.3)
	50	5	78.0 e	5.5 bc	66.4 fg	0.0	0.0	2.1 def (4.1)	0.1 (0.3)
	50	15	82.0 e	2.3 b	69.2 g	0.0	0.0	0.4 abc (1.0)	0.0 (0.1)
	50	20	58.0 cd	3.1 b	52.0 de	0.0	0.0	1.3 bcdef (2.6)	0.2 (0.5)
	50	30	43.0 b	3.1 b	14.9 b	0.0	0.0	0.8 abc (1.6)	0.2 (0.5)
	60	5	0.6 a	0.0 a	0.4 a	0.0	0.0	1.0 abcde (2.0)	0.2 (0.5)
	60	15	0.6 a	0.0 a	0.2 a	0.0	0.0	0.1 a (0.3)	0.2 (0.5)
	60	20	0.6 a	0.0 a	0.4 a	0.0	0.0	0.2 ab (0.5)	0.2 (0.5)
	60	30	0.6 a	0.0 a	0.4 a	0.0	0.0	0.4 abc (1.0)	0.1 (0.3)
*Probability*^*d*^	* *	* *	*<0*.*001*	*<0*.*001*	*<0*.*001*	*0*.*17*	*0*.*68*	*<0*.*001*	*0*.*55*
HW2	RT	15	43.4 b	21.7 c	38.0 b	-	-	-	-
	40	15	59.4 c	14.0 c	63.0 c	-	-	-	-
	50	15	80.7 d	6.7 b	66.7 c	-	-	-	-
	60	15	0.0 a	0.0 a	0.0 a	-	-	-	-
	70	15	0.0 a	0.0 a	0.0 a	-	-	-	-
*Probability*^*d*^	* *	* *	*<0*.*001*	*<0*.*001*	*<0*.*001*	* *	* *	* *	* *
HW3	RT	0	44.6 cd	17.0 d	56.6 bc	-	-	-	-
	30	5	35.6 c	16.4 d	57.0 c	-	-	-	-
	30	15	51.3 de	16.7 d	44.6 bc	-	-	-	-
	30	30	62.0 ef	14.4 cd	57.0 c	-	-	-	-
	40	5	58.3 ef	15.4 cd	54.3 bc	-	-	-	-
	40	15	67.6 f	11.7 cd	44.0 bc	-	-	-	-
	40	30	70.6 f	13.7cd	52.0 bc	-	-	-	-
	50	5	68.6 f	9.7 bcd	48.0 bc	-	-	-	-
	50	15	68.3 f	7.0 bc	53.6 bc	-	-	-	-
	50	30	50.6 de	4.0 ab	43.3 b	-	-	-	-
	60	5	4.3 b	0.4 a	1.0 a	-	-	-	-
	60	15	0.0 a	0.4 a	0.0 a	-	-	-	-
	60	30	0.0 a	0.4 a	0.0 a	-	-	-	-
*Probability*^*d*^			*<0*.*001*	*<0*.*001*	*<0*.*001*				

^a^ Values were obtained from 100 seeds plated onto water agar.

^b^ Values were obtained from 100 plants in three-week-old seedlings for both emergence and visual disease symptoms.

^c^ Results were obtained from 15 subsamples of 10 four-week-old seedlings from individual seed treatment by PCR. In PCR, weak positive amplifications were also considered as a positive detection [5]. Values in parentheses indicate the estimated pathogen transmission incidence (%) of based on the testing procedure of Gibbs and Gower [33].

^d^ Treatment differences were analysed via logistic regression. Germination, contamination, emergence and symptom incidences were analysed using number of positive seeds/seedlings out of the total number assessed. Incidence of *P*. *somniferi* and *P*. *meconopsidis* were analysed using the number of positive PCR reactions out of the total number of tests conducted. Post hoc comparisons were undertaken using least square means estimates with a Tukey correction for multiple pairwise comparisons. Treatment estimates sharing a lowercase letter within an experiment are not significantly different from each other (*P* ≥ 0.05).

^e^ RT, ambient room temperature (18–22°C)

### Aerated steam treatment

Seed germination was improved by aerated stem treatment for 30 min or less at temperatures of 50°C or less (Table 5). Treatment up to 60°C for 15 min or less also either significantly improved or did not reduce germination rates relative to the control across all experiments. However, treatment for 30 min or more at 60°C or higher was typically detrimental to germination rate. When treated with 55°C or higher for 15 min or longer seed contamination consistently demonstrated significant reductions, relative to the control. At lower temperatures effects on contamination were less consistent across experiments. No positive effects of aerated steam treatment were detected in ST1 and ST3. However, in ST2, 60°C for 10 and 15 min, 50°C for 30 min or less and 40°C for all durations significantly improved germination (Table 5). Incidence of both *P*. *somniferi* and *P*. *meconopsidis* in germinated seedlings was significantly reduced by treatment at 60°C for all durations, while *P*. *somniferi* was also reduced at 50°C for 60 min and *P*. *meconopsidis* at 40°C for either 30 or 60 min. No significant differences in symptom incidence were observed.

**Table 5:** The effect of aerated steam treatment at various temperature and time on the opium poppy seed health and controlling poppy downy mildew.

Experiment	Temperature (°C)	Time (min)	Germination (%) ^a^	Contamination (%) ^a^	Emergence (%) ^b^	Systemic symptom incidence (%) ^b^	Localised symptom incidence (%) ^b^	*P*. *somniferi* incidence in seedlings ^c^	*P*. *meconopsidis* incidence in seedlings ^c^
ST1	RT^e^	0	49.5 cd	18.8 f	46.0 c	0.6	0.0	6.8 c (4.1)	0.7 b (1.3)
	40	15	61.0 d	17.6 ef	55.6 c	0.3	0.0	6.3 bc (3.6)	0.1 ab (0.3)
	40	30	63.6 d	13.6 def	53.3 c	0.0	0.0	5.0 bc (2.6)	0.0 a (0.0)
	40	60	41.3 c	10.3 cde	31.3 b	0.3	0.0	3.3 abc (1.7)	0.0 a (0.0)
	50	15	62.6 d	12.6 def	51.0 c	0.3	0.0	7.3 bc (4.4)	0.1 ab (0.4)
	50	30	40.3 bc	7.0 bcd	34.3 b	0.0	0.0	3.3 abc (1.7)	0.1 ab (0.4)
	50	60	28.6 b	7.3 bcd	26.0 b	0.0	0.0	1.0 a (0.4)	0.1 ab (0.4)
	60	15	62.6 d	4.0 bc	53.6 c	0.0	0.0	3.0 ab (1.4)	0.0 a (0.0)
	60	30	28.6 b	2.6 ab	28.0 b	0.0	0.0	1.0 a (0.4)	0.0 a (0.0)
	60	60	0.6 a	0.0 a	0.3 a	0.0	0.0	0.3 a (0.1)	0.0 a (0.0)
*Probability*^*d*^	* *	* *	*<0*.*001*	*<0*.*001*	*<0*.*001*	*0*.*49*	*1*.*00*	*<0*.*001*	*<0*.*001*
ST2	RT	0	56.0 bc	13.6 b	43.5 c	-	-	-	-
	40	10	72.3 d	10.0 b	68.3 e	-	-	-	-
	40	15	67.3 d	13.0 b	64.6 de	-	-	-	-
	40	30	68.8 d	10.0 b	66.3 de	-	-	-	-
	40	40	63.8 cd	12.0 b	62.0 de	-	-	-	-
	50	10	69.7 d	10.0 b	63.0 de	-	-	-	-
	50	15	67.0 d	9.3 b	62.3 de	-	-	-	-
	50	30	67.3 d	8.3 b	58.6 de	-	-	-	-
	50	40	46.6 b	9.0 b	26.3 b	-	-	-	-
	60	10	69.6 d	7.6 b	62.0 de	-	-	-	-
	60	15	64.6 cd	1.0 a	54.6 d	-	-	-	-
	60	30	24.0 a	0.0 a	14.6 a	-	-	-	-
	60	40	25.0 a	0.0 a	11.0 a	-	-	-	-
*Probability*^*d*^	* *	* *	*<0*.*001*	*<0*.*001*	*<0*.*001*	* *	* *	* *	* *
ST3	RT	0	62.0 b	0.2 c	48.7 c	-	-	-	-
	40	15	70.4 bc	0.2 c	55.0 c	-	-	-	-
	45	15	77.4 c	0.1 bc	52.4 c	-	-	-	-
	50	15	59.7 b	0.1 b	49.4 c	-	-	-	-
	55	15	63.0 b	0.1 b	55.4 c	-	-	-	-
	60	15	60.7 b	0.0 a	55.7 c	-	-	-	-
	65	15	59.4 b	0.0 a	13.7 b	-	-	-	-
	70	15	17.4 a	0.0 a	0.00 a	-	-	-	-
*Probability*^*d*^	* *	* *	*<0*.*001*	*<0*.*001*	*<0*.*001*	* *	* *	* *	* *

^a^ Values were obtained from 100 seeds plated onto water agar.

^b^ Values were obtained from 100 plants in three-week-old seedlings for both emergence and visual disease symptoms.

^c^ Results were obtained from 15 subsamples of 10 four-week-old seedlings from individual seed treatment by PCR. In PCR, weak positive amplifications were also considered as a positive detection [5]. Values in parentheses indicate the estimated pathogen transmission incidence (%) of based on the testing procedure of Gibbs and Gower [33]

^d^ Treatment differences were analysed via logistic regression. Germination, contamination, emergence and symptom incidences were analysed using number of positive seeds/seedlings out of the total number assessed. Incidence of *P*. *somniferi* and *P*. *meconopsidis* were analysed using the number of positive PCR reactions out of the total number of tests conducted. Post hoc comparisons were undertaken using least square means estimates with a Tukey correction for multiple pairwise comparisons. Treatment estimates sharing a lowercase letter within an experiment are not significantly different from each other (*P* ≥ 0.05).

^e^RT, ambient room temperature (18–22°C)

### Treatment protocol comparison

Across both treatment comparison experiments, the effect of single treatments on seed germination was consistent (Table 1). All rates of hypochlorite and electrolysis water significantly improved germination relative to the controls of each experiment. Furthermore, hot water at 50°C and aerated steam at 50 or 60°C significantly improved germination in both experiments. In PC1, highest germination rates were observed when seed was treated with 400 ppm electrolysis water (91%; Table 6). In PC2, the highest germination rates were achieved with 2% hypochlorite. Except for hot water (40°C) followed by aerated steam (60°C) in PC2, no significant improvements in germination rate relative to the first treatment in the combination were observed. In the case of hot water and aerated steam, this treatment was not significantly better than aerated steam alone.

**Table 6:** Comparison and efficacy of four seed treatment on enhancing opium poppy seed health and controlling poppy downy mildew.

Experiment	Treatment	Incubation Temperature (°C)	Hypochlorite (%)	Hypocholorus acid (ppm)	Time (min)	Germination (%) ^a^	Contamination (%) ^a^	Emergence (%) ^b^	*P*. *somniferi* incidence in seedlings ^c^	*P*. *meconopsidis* incidence in seedlings ^c^
Protocol comparison (PC1)	Untreated	RT^e^	-	-	-	49.4 bc	17.3 d	60.5 cde	5.3 de (8.00)	0.9 (1.7)
	Hypochlorite solution	5	2	-	5	86.7 efg	2.3 ab	68.0 def	0.6 abc (1.34)	0.0 (0.0)
	Electrolysis water	RT	-	200	15	80.0 cdef	8.3 bc	65.3 cdef	3.3 de (5.67)	0.0 (0.0)
		RT	-	400	15	91.4 g	2.3 ab	70.6 efg	0.4 ab (1.00)	0.0 (0.0)
	Hot water	40	-	-	15	37.7 b	12.6 cd	55.0 cd	6.3 e (9.34)	0.0 (0.0)
		50	-	-	15	73.4 d	7.6 bc	54.6 c	4.6 de (2.60)	0.3 (0.7)
		60	-	-	15	0.0 a	0.6 a	0.0 a	0.0 a (0.00)	0.0 (0.0)
		70	-	-	15	0.0 a	0.6 a	0.0 a	0.0 a (0.00)	0.0 (0.0)
	Aerated steam	50	-	-	15	85.4 defg	12.3 cd	76.67 fg	4.3 de (7.00)	0.0 (0.0)
		60	-	-	-	76.7 cde	10.0 cd	73.3 fg	2.9 cde (5.00)	0.2 (0.3)
	Electrolysis water + Hot water	RT+ 40	-	200	15 (HOCI), 15 (Hot water)	76.0 cd	9.3 cd	12.0 b	2.2 bcd (4.00)	0.0 (0.0)
	Electrolysis water + Hot water	RT + 60	-	200	15 + 15	0.0 a	2.3 ab	0.0 a	0.0 a (0.00)	0.0 (0.0)
	Electrolysis water + Aerated steam	RT + 60	-	200	15 + 15	87.7 fg	8.0 bc	81.6 g	0.5 ab (1.00)	0.0 (0.0)
*Probability*^*d*^		* *	* *	* *	* *	*<0*.*001*	*<0*.*001*	*<0*.*001*	*<0*.*001*	*0*.*70*
Protocol comparison (PC2)	Untreated	RT	-	-	-	71.2 c	24.5 g	40.4 cd	3.0 c (2.18)	0.2 (0.3)
	Hypochlorite solution	RT	1	-	5	87.8 de	4.4 cd	82.9 gh	0 .0 a (0.00)	0.0 (0.0)
		RT	2	-	5	96.7 g	1.9 abc	90.9 i	0.2 a (0.08)	0.0 (0.0)
		RT	4	-	5	89.0 de	4.4 cd	86.7 hi	0.00 a (0.00)	0.0 (0.0)
	Electrolysis water	RT	-	100	15	92.4 defg	11.7 ef	48.0 de	0.7 abc (0.3)	0.0 (0.0)
		RT	-	200	15	96.0 fg	5.5 cd	97.2 j	0.3 a (0.16)	0.0 (0.0)
		RT	-	400	15	95.4 fg	0.4 ab	96.0 j	0.2 a (0.08)	0.0 (0.0)
	Hot water	30	-	-	15	60.7 b	26.2 g	45.0 cde	2.7 bc (1.51)	0.0 (0.0)
		40	-	-	15	74.0 c	18.4 fg	36.0 c	1.7 abc (0.98)	0.0 (0.0)
		50	-	-	15	86.9 d	9.0de	71.2 f	0.5 ab (0.24)	0.0 (0.0)
		60	-	-	15	0.6 a	0.0 a	0.0 a	0.0 a (0.00)	0.0 (0.0)
	Aerated steam	40	-	-	15	79.0 c	15.9 f	72.0 f	1.5 abc (0.8)	0.0 (0.0)
		50	-	-	15	87.8 de	8.2 de	74.7 fg	0.8 abc (0.41)	0.0 (0.0)
		60	-	-	15	92.5 defg	5.4 cd	89.2 hi	0.5 ab (0.24)	0.0 (0.0)
		70	-	-	15	9.0 a	0.0 a	0.0 a	0.0 a (0.00)	0.0 (0.0)
	Hypochlorite solution + Electrolysis water	RT + RT	2	200	5 + 15	93.5 efg	0.9 ab	75.5 fg	0.0 a (0.00)	0.0 (0.0)
	Hypochlorite solution + Hot water	RT + 40	2	-	5 + 15	96.5 g	2.0 abc	90.2 i	0.0 a (0.00)	0.0 (0.0)
	Hypochlorite solution + Aerated steam	RT + 60	2	-	5 + 15	91.0 def	2.9 bc	43.9 cd	0.0 a (0.00)	0.0 (0.0)
	Electrolysis water + Aerated steam	RT + 60	-	200	15 + 15	70.8 c	0.90 ab	26.2 b	0.0 a (0.00)	0.0 (0.0)
	Electrolysis water + Hot water	RT + 40	-	200	15 + 15	89.7 de	5.4 cd	54.9 e	0.5 ab (0.25)	0.0 (0.0)
	Hot water + Aerated steam	40 + 60	-	-	15 + 15	88.9 de	4.4 cd	23.5 b	0.2 a (0.08)	0.0 (0.0)
*Probability*^*d*^		* *	* *	* *	* *	*<0*.*001*	*<0*.*001*	*<0*.*001*	*<0*.*001*	*0*.*90*

^a^ Values were obtained from 100 seeds plated onto water agar.

^b^ Values were obtained from 100 plants in three-week-old seedlings for both emergence and visual disease symptoms.

^c^ Results were obtained from 15 subsamples of 10 four-week-old seedlings from individual seed treatment by PCR. In PCR, weak positive amplifications were also considered as a positive detection [5]. Values in parentheses indicate the estimated pathogen transmission incidence (%) of based on the testing procedure of Gibbs and Gower [33]

^d^ Treatment differences were analysed via logistic regression. Germination, contamination, emergence and symptom incidences were analysed using number of positive seeds/seedlings out of the total number assessed. Incidence of *P*. *somniferi* and *P*. *meconopsidis* were analysed using the number of positive PCR reactions out of the total number of tests conducted. Post hoc comparisons were undertaken using least square means estimates with a Tukey correction for multiple pairwise comparisons. Treatment estimates sharing a lowercase letter within an experiment are not significantly different from each other (*P* ≥ 0.05).

^e^ RT, ambient room temperature (18–22°C)

Hypochlorite and electrolysis water also significantly reduced seed contamination rates in both experiments at all concentrations. The effects of aerated steam and hot water was less consistent across experiments. However, contamination was lowest when treated with hot water at 60°C in both experiments. Combining electrolysis water, hypochlorite or aerated steam with other treatments did not reduce contamination below that of those treatments alone.

In PC1, the mean germination rate was significantly greater than the untreated control in all treatments except the combined 200 ppm HOCI for 15 min & hot water at 60°C for 15 min, hot water at 40°C, 60°C and 70°C for 15 min.

Seedling emergence was only significantly greater than the control when treated with aerated steam at 50 or 60°C for 15 min, and when electrolysis water was used with aerated steam at 60°C in PC1 (Table 6). In the latter case, aerated steam in combination with electrolysis water was not significantly different from aerated steam at 60°C alone. In PC2 all hypochlorite concentrations, 200 and 400 ppm electrolysis water, hot water at 50°C and aerated steam at 40, 50 and 60°C significantly increased emergence. No combined treatment resulted in a significant increase in emergence above that of its constituent treatments.

In PC1, incidence of *P*. *somniferi* in control seedlings was 8.0% (Table 6). Treatment with hypochlorite (2%), electrolysis water (400 ppm) and hot water (60 and 70°C) all significantly reduced incidence of *P*. *somniferi* below the control. Electrolysis water (200 ppm) in combination with aerated steam (60°C) also significantly reduced *P*. *somniferi* incidence relative to both the control and each of the individual constituent treatments. In PC2, hypochlorite (1, 2, and 4%), electrolysis water (100, 200 and 400 ppm), hot water (50 and 60°C) and aerated steam (60 and 70°C) all significantly reduced *P*. *somniferi* incidence relative to the 3.0% incidence observed in the control. All combination treatments also significantly reduced *P*. *somniferi* incidence, but none were significant reductions relative to either one or both of their individual constituents. No *P*. *somniferi* infected seedlings were detected when seed was treated with hypochlorite at 1 or 4%, or 2% in combination with another treatment, nor when treated with electrolysis water in combination with hot water at 40°C. Incidence of *P*. *meconopsidis* in seedlings was low (≤1.7%) for all treatments across both experiments and no significant treatment effects were detected.

## Discussion

This study was undertaken to examine the potential of physiochemical seed treatments to manage seed-borne transmission of downy mildew in opium poppy. Many *Peronospora* spp. are known to be seed-borne, including those infecting poppy [5, 9, 36, 37]. Seed-borne transmission of disease can initiate inocula foci from which secondary spread occurs [5, 8, 10]. Furthermore, seed-borne infestations can be transported across great distances by anthropogenic mean allowing the introduction of pathogens to regions with no previous history of the disease.

Hypochlorite treatments have been commonly used as sanitisers and as seed disinfectants [13, 38]. In the range of hypochlorite treatments tested, 2% hypochlorite for 5 min gave the greatest seed germination and emergence, whilst minimising microbial contamination and reducing *P*. *somniferi* transmission to seedlings. This suggests hypochlorite at this rate could be an effective seed treatment to both reduce disease risks and improve seed health. Similar outcomes have been also reported in barley and lettuce to eradicate *Alternaria* sp., and *Xanthomonas* sp. from seed lots respectively [38–40]. However, hypochlorite treatment requires careful removal of the chemical by multiple water rinses, otherwise the residues on the seed coat increase the risk of damage to seed lines during emergence [13]. Also handling of hypochlorite solutions poses potential risk to users as it is highly caustic and produces dangerous volatiles. Thus, for commercial use, hypochlorite-based seed treatments need careful attention to handling procedure and seed processing to minimise risks.

Electrolysis water is widely used in food industries, healthcare environments and in agriculture as a sanitiser [41, 42]. Recent studies have shown that electrolysis water application can reduce the spore germination of species in the genera *Botrytis*, *Fusarium*, *Penicillium*, *Cladosporium*, *Helminthosporium* and *Phytophthora* [43, 44]. In this study, poppy seeds treated with 400 ppm of electrolysis water for 15 min had improved germination, decreasing microbial contamination and a significant reduction in the transmission of *P*. *somniferi*. In contrast to hypochlorite treatments, electrolysis water does not require rinsing as the breakdown products are sodium chloride and water, and it poses less risk to the operator [41, 45].

Conventional heating methods, such as hot water and aerated steam, have been reported as a valuable for controlling many seed-borne pathogens. These include black leg and black rot in cabbage caused by *Phoma lingam* and *Xanthomonas campestris* pv. *campestris*, respectively [46, 47]. However, these were less efficacious than chlorine-based treatments in this study. The most suitable temperature and duration of hot water was 50°C for 15 min with higher temperatures (>50°C) and longer durations (>20 min) having a significant detrimental effect on germination and emergence suggesting loss of seed viability. In addition, seed treated with hot water often results in a reduction in viability if not stored appropriately, as the seed coat can form cracks during treatment [48]. Long term viability of poppy seeds post treatment was not assessed in this study. However, this should be evaluated prior to commercial adoption of any treatment protocol by the poppy industry. Aerated steam treatment at 60°C for 15 min was found sufficient to enhance the seed health and minimise transmission of *Peronospora* spp. in seedlings. However, as with hot water treatment, increased temperatures and durations had a negative effect on seed performance.

An additional benefit of seed treatments observed in this study was the reduction in fungal contaminants other than *Peronospora* spp.. While, not the focus of this study, poppy fire, caused by *Alternaria penicillata* and *A*. *papavericola* [49], is another seed-borne disease of opium poppy [50–52]. Poppy fire is known to be present in Australian crops [53], however the significance of the disease for Australian production is currently unknown. Whether or not the co-occurrence of reduced seed fungal contamination and increased seedling emergence following physiochemical treatment in this study is due to poppy fire contamination of seed bears further study.

Physical seed treatment methods can also enhance seed viability. Physical treatments can break the dormancy of the seed and that enhance the uniformity in germination and emergence [54–56]. Seed scarification methods, including chlorine and temperature-based treatments, can soften the seed coat, increasing the seed gas exchange rate and moisture content [54–57]. Our standardized treatments from these both methods provide satisfactory germination, emergence and effective microbial suppression. Treating poppy seeds either with 2% hypochlorite solution for 5 min or 400 ppm electrolysis water results in higher germination rates and adequate pathogen elimination compared to temperature-based treatments. This could be because chlorine-based treatments enhance the water regulation within the seed [55] and therefore, provide more synchronized germination and emergence.

A major driver for the evaluation of non-chemical fungicide seed treatments in this work is the perceived risk of fungicide resistance development. In season fungicide applications provide the principle mechanism for in season disease management for the Australian poppy industry [58]. However, such reliance carries with it the risk of resistance development. While fungicide resistance has not been detected in the poppy pathogens, it has occurred in other downy mildew species with resistance to metalaxyl [59, 60] and mefenoxam [61, 62] recorded. Thus, to mitigate the risk of this occurring, seed treatment options based on alternatives to chemical fungicides as seen as beneficial for poppy production.

In conclusion, in this study we have demonstrated the benefits of four physiochemical seed treatments for enhancing the poppy seed health and performance, and minimising transmission of *P*. *somniferi* and *P*. *meconopsidis*. Of the four physio-chemical treatments, the chlorine-based hypochlorite and electrolysis water treatments appeared superior to be temperature-based treatments in controlling pathogen transmission whilst retaining seed performance. Combining multiple treatments in series was not observed to give an advantage over individual treatments. We recommend consideration of hypochlorite or electrolysis water seed treatments for treatment of commercial poppy seed lots to minimise downy mildew transmission and improve seed performance.
