# Supplementary material for: Effect of physio-chemical seed treatments on opium poppy downy mildews caused by Peronospora meconopsidis and P. somniferi
Source: PLoS One. 2020 Apr 10;15(4):e0230801. doi: 10.1371/journal.pone.0230801 (PMC7147767; doi:10.1371/journal.pone.0230801)
Supplement: Attachment — Submitted filename: Response to Reviewers.docx [file pone.0230801.s001.docx]

Tasmanian Institute of Agriculture

New Town Research Laboratories

13 St Johns Avenue New Town Tasmania 7008 Australia

Telephone +61 3 6226 6381

www.tia.tas.edu.au

06/03/2020

**Re: M/S: PONE-D-19-20356 “Effect of physio-chemical seed treatments on opium poppy downy mildews caused by *Peronospora meconopsidis* and *P. somniferi*.”**

Dear Dr Sabrina Sarrocco,

Thank you and the reviewer for your helpful comments. We have considered each and made the suggested changes were appropriate unless otherwise stated below.

We hope the manuscript is now acceptable for publication

Kind Regards,

(On behalf of all the authors)

Dr. Tamilarasan Thangavel

Corresponding Author

**Response to Editor**

1. Please ensure that your manuscript meets PLOS ONE's style requirements, including those for file naming. The PLOS ONE style templates can be found at
<http://www.journals.plos.org/plosone/s/file?id=wjVg/PLOSOne_formatting_sample_main_body.pdf> and <http://www.journals.plos.org/plosone/s/file?id=ba62/PLOSOne_formatting_sample_title_authors_affiliations.pdf>
**Response:** We have checked and ensured adherence to PLOS ONE’s style

2. Thank you for stating the following financial disclosure:

"The funders had no role in study design, data collection and analysis, decision to publish, or preparation of the manuscript."

a. Please provide an amended Funding Statement that declares *all* the funding or sources of support received during this specific study (whether external or internal to your organization) as detailed online in our guide for authors at <http://journals.plos.org/plosone/s/submit-now>.

**Response:** We have mentioned the funding statement in the cover letter.

b. Please state what role the funders took in the study. If any authors received a salary from any of your funders, please state which authors and which funder. If the funders had no role, please state: "The funders had no role in study design, data collection and analysis, decision to publish, or preparation of the manuscript."

**Response:** This sentence has been included in the cover letter: “The funders had no role in the study design, data collection and analysis, decision to publish or preparation of the manuscript.”

c. Please include your amended statements within your cover letter; we will change the online

**Response: We have amended the cover** letter (attached) as requested.

**Response to Reviewer**

**Reviewer #1:**

1. Title should have been " Effect of physio-chemical seed treatments on opium poppy downy mildews caused by *Peronospora meconopsidis* and *P. somniferi.*

**Response: Accept**

We have modified the title as suggested.

2. Please write room and ambient temperatures in entire manuscript

**Response: Accept**

We have changed from “room and ambient temperature” to ambient room temperature, and temperature range that this refers to is now defined (see below) where it is appropriate in the manuscript.

e.g.

Line 88 : Seed was stored at ambient room temperature (18-22°C) in the dark before use.

Line 111 : The controls for steam treatment studies were seed exposed to ambient room air (18-22°C)

3. Please pay attention to grammar.

**Response: Accept**

We have thoroughly checked the manuscript for grammatical and typo errors, and we made some changes that are indicated in the manuscript.

4. Revise table 1, expand codes used in column 1,
   column 2 should be Treatments and concentrations. Hence move data from 4 and 5 into column 2 Column 3 should be Incubation Temp (C) and duration (min). Hence move data from column 6 into column 3.

**Response: Accept**

We have altered Table 1 taking into consideration the reviewer’s comments. He revised table now has the following columns: 1) Experiment code, 2 ) Treatment type, 3) Incubation time, 4) Chlorine concertation and 5) Incubation temperature. We have elected not to combine temperature and duration into a single column as we feel that the mixing of different parameters in this manner results into a more confusing presentation of the data.
