# Supplementary material for: Effect of physio-chemical seed treatments on opium poppy downy mildews caused by Peronospora meconopsidis and P. somniferi
Source: PLoS One. 2020 Apr 10;15(4):e0230801. doi: 10.1371/journal.pone.0230801 (PMC7147767; doi:10.1371/journal.pone.0230801)
Supplement: Peer review history

# Peer review: Decision Letter 0

**DOI:** 10.1371/journal.pone.0230801.r001

**Authors:** Sabrina Sarrocco

26 Feb 2020

PONE-D-19-20356

Efficacy of seed treatments for disinfestation of Peronospora spp. from Papaver somniferum seeds and control of seed-borne poppy downy mildew

PLOS ONE

Dear Dr Thangavel,

Thank you for submitting your manuscript to PLOS ONE. After careful consideration, we feel that it has merit but does not fully meet PLOS ONE’s publication criteria as it currently stands. Therefore, we invite you to submit a revised version of the manuscript that addresses the points raised during the review process.

We apologize for the delay in ending the revision process, this due to difficulties in finding available reviewers for managing your paper.

We would appreciate receiving your revised manuscript by Apr 11 2020 11:59PM. When you are ready to submit your revision, log on to https://www.editorialmanager.com/pone/ and select the 'Submissions Needing Revision' folder to locate your manuscript file.

If you would like to make changes to your financial disclosure, please include your updated statement in your cover letter.

To enhance the reproducibility of your results, we recommend that if applicable you deposit your laboratory protocols in protocols.io, where a protocol can be assigned its own identifier (DOI) such that it can be cited independently in the future. For instructions see: http://journals.plos.org/plosone/s/submission-guidelines#loc-laboratory-protocols

Please include the following items when submitting your revised manuscript:

- A rebuttal letter that responds to each point raised by the academic editor and reviewer(s). This letter should be uploaded as separate file and labeled 'Response to Reviewers'.
- A marked-up copy of your manuscript that highlights changes made to the original version. This file should be uploaded as separate file and labeled 'Revised Manuscript with Track Changes'.
- An unmarked version of your revised paper without tracked changes. This file should be uploaded as separate file and labeled 'Manuscript'.

Please note while forming your response, if your article is accepted, you may have the opportunity to make the peer review history publicly available. The record will include editor decision letters (with reviews) and your responses to reviewer comments. If eligible, we will contact you to opt in or out.

We look forward to receiving your revised manuscript.

Kind regards,

Sabrina Sarrocco

Academic Editor

PLOS ONE

Journal Requirements:

When submitting your revision, we need you to address these additional requirements.

1. Please ensure that your manuscript meets PLOS ONE's style requirements, including those for file naming. The PLOS ONE style templates can be found at

http://www.journals.plos.org/plosone/s/file?id=wjVg/PLOSOne_formatting_sample_main_body.pdf and http://www.journals.plos.org/plosone/s/file?id=ba62/PLOSOne_formatting_sample_title_authors_affiliations.pdf

2. Thank you for stating the following financial disclosure:

"The funders had no role in study design, data collection and analysis, decision to publish, or preparation of the manuscript."

1. Please provide an amended Funding Statement that declares *all* the funding or sources of support received during this specific study (whether external or internal to your organization) as detailed online in our guide for authors at http://journals.plos.org/plosone/s/submit-now.  

1. Please state what role the funders took in the study.  If any authors received a salary from any of your funders, please state which authors and which funder. If the funders had no role, please state: "The funders had no role in study design, data collection and analysis, decision to publish, or preparation of the manuscript."

Please include your amended statements within your cover letter; we will change the online submission form on your behalf.

[Note: HTML markup is below. Please do not edit.]

Reviewers' comments:

Reviewer's Responses to Questions

**Comments to the Author**

1. Is the manuscript technically sound, and do the data support the conclusions?

The manuscript must describe a technically sound piece of scientific research with data that supports the conclusions. Experiments must have been conducted rigorously, with appropriate controls, replication, and sample sizes. The conclusions must be drawn appropriately based on the data presented. 

Reviewer #1: Yes

**********

2. Has the statistical analysis been performed appropriately and rigorously? 

Reviewer #1: Yes

**********

3. Have the authors made all data underlying the findings in their manuscript fully available?

The PLOS Data policy requires authors to make all data underlying the findings described in their manuscript fully available without restriction, with rare exception (please refer to the Data Availability Statement in the manuscript PDF file). The data should be provided as part of the manuscript or its supporting information, or deposited to a public repository. For example, in addition to summary statistics, the data points behind means, medians and variance measures should be available. If there are restrictions on publicly sharing data—e.g. participant privacy or use of data from a third party—those must be specified.

Reviewer #1: Yes

**********

4. Is the manuscript presented in an intelligible fashion and written in standard English?

PLOS ONE does not copyedit accepted manuscripts, so the language in submitted articles must be clear, correct, and unambiguous. Any typographical or grammatical errors should be corrected at revision, so please note any specific errors here.

Reviewer #1: Yes

**********

5. Review Comments to the Author

Please use the space provided to explain your answers to the questions above. You may also include additional comments for the author, including concerns about dual publication, research ethics, or publication ethics. (Please upload your review as an attachment if it exceeds 20,000 characters)

Reviewer #1: Title should have been " Effect of physio-chemical seed treatments on opium poppy downy mildews caused by Peronospora meconopsidis and P. somniferi.

Please write room and ambient temperatures in entire manuscript

Please pay attention to grammar.

Revise table 1, expand codes used in column 1,

column 2 should be Treatments and concentrations. Hence move data from 4 and 5 in to column 2

Column 3 should be Incubation Temp (C) and duration (min). Hence move data from column 6 in to column 3.

**********

6. PLOS authors have the option to publish the peer review history of their article (what does this mean?). If published, this will include your full peer review and any attached files.

If you choose “no”, your identity will remain anonymous but your review may still be made public.

**Do you want your identity to be public for this peer review?** For information about this choice, including consent withdrawal, please see our Privacy Policy.

Reviewer #1: No

[NOTE: If reviewer comments were submitted as an attachment file, they will be attached to this email and accessible via the submission site. Please log into your account, locate the manuscript record, and check for the action link "View Attachments". If this link does not appear, there are no attachment files to be viewed.]

While revising your submission, please upload your figure files to the Preflight Analysis and Conversion Engine (PACE) digital diagnostic tool, https://pacev2.apexcovantage.com/. PACE helps ensure that figures meet PLOS requirements. To use PACE, you must first register as a user. Registration is free. Then, login and navigate to the UPLOAD tab, where you will find detailed instructions on how to use the tool. If you encounter any issues or have any questions when using PACE, please email us at figures@plos.org. Please note that Supporting Information files do not need this step.

# Author comment: Author response to Decision Letter 0

**DOI:** 10.1371/journal.pone.0230801.r002

6 Mar 2020

Re: M/S: PONE-D-19-20356 “Effect of physio-chemical seed treatments on opium poppy downy mildews caused by Peronospora meconopsidis and P. somniferi.” 

Dear Dr Sabrina Sarrocco,

Thank you and the reviewer for your helpful comments. We have considered each and made the suggested changes were appropriate unless otherwise stated below.

We hope the manuscript is now acceptable for publication

Kind Regards,

(On behalf of all the authors)

Dr. Tamilarasan Thangavel

Corresponding Author

 

Response to Editor

1. Please ensure that your manuscript meets PLOS ONE's style requirements, including those for file naming. The PLOS ONE style templates can be found at

http://www.journals.plos.org/plosone/s/file?id=wjVg/PLOSOne_formatting_sample_main_body.pdf and http://www.journals.plos.org/plosone/s/file?id=ba62/PLOSOne_formatting_sample_title_authors_affiliations.pdf

Response: We have checked and ensured adherence to PLOS ONE’s style

2. Thank you for stating the following financial disclosure:

"The funders had no role in study design, data collection and analysis, decision to publish, or preparation of the manuscript."

a. Please provide an amended Funding Statement that declares *all* the funding or sources of support received during this specific study (whether external or internal to your organization) as detailed online in our guide for authors at http://journals.plos.org/plosone/s/submit-now.

Response: We have mentioned the funding statement in the cover letter. 

b. Please state what role the funders took in the study. If any authors received a salary from any of your funders, please state which authors and which funder. If the funders had no role, please state: "The funders had no role in study design, data collection and analysis, decision to publish, or preparation of the manuscript."

Response: This sentence has been included in the cover letter: “The funders had no role in the study design, data collection and analysis, decision to publish or preparation of the manuscript.”

c. Please include your amended statements within your cover letter; we will change the online 

Response: We have amended the cover letter (attached) as requested.

Response to Reviewer

Reviewer #1: 

1. Title should have been " Effect of physio-chemical seed treatments on opium poppy downy mildews caused by Peronospora meconopsidis and P. somniferi.

Response: Accept 

We have modified the title as suggested.

2. Please write room and ambient temperatures in entire manuscript

Response: Accept 

We have changed from “room and ambient temperature” to ambient room temperature, and temperature range that this refers to is now defined (see below) where it is appropriate in the manuscript.

e.g.

Line 88 : Seed was stored at ambient room temperature (18-22°C) in the dark before use.

Line 111 : The controls for steam treatment studies were seed exposed to ambient room air (18-22°C)

3. Please pay attention to grammar.

Response: Accept 

We have thoroughly checked the manuscript for grammatical and typo errors, and we made some changes that are indicated in the manuscript.

4. Revise table 1, expand codes used in column 1,

column 2 should be Treatments and concentrations. Hence move data from 4 and 5 into column 2 Column 3 should be Incubation Temp (C) and duration (min). Hence move data from column 6 into column 3.

Response: Accept 

We have altered Table 1 taking into consideration the reviewer’s comments. He revised table now has the following columns: 1) Experiment code, 2 ) Treatment type, 3) Incubation time, 4) Chlorine concertation and 5) Incubation temperature. We have elected not to combine temperature and duration into a single column as we feel that the mixing of different parameters in this manner results into a more confusing presentation of the data.

Attachment Submitted filename: Response to Reviewers.docx Click here for additional data file.

# Editor report: Decision Letter 1

**DOI:** 10.1371/journal.pone.0230801.r003

**Authors:** Sabrina Sarrocco

10 Mar 2020

Effect of physio-chemical seed treatments on opium poppy downy mildews caused by Peronospora meconopsidis and P. somniferi.

PONE-D-19-20356R1

Dear Dr. Thangavel,

We are pleased to inform you that your manuscript has been judged scientifically suitable for publication and will be formally accepted for publication once it complies with all outstanding technical requirements.

Within one week, you will receive an e-mail containing information on the amendments required prior to publication. When all required modifications have been addressed, you will receive a formal acceptance letter and your manuscript will proceed to our production department and be scheduled for publication.

Shortly after the formal acceptance letter is sent, an invoice for payment will follow. To ensure an efficient production and billing process, please log into Editorial Manager at https://www.editorialmanager.com/pone/, click the "Update My Information" link at the top of the page, and update your user information. If you have any billing related questions, please contact our Author Billing department directly at authorbilling@plos.org.

If your institution or institutions have a press office, please notify them about your upcoming paper to enable them to help maximize its impact. If they will be preparing press materials for this manuscript, you must inform our press team as soon as possible and no later than 48 hours after receiving the formal acceptance. Your manuscript will remain under strict press embargo until 2 pm Eastern Time on the date of publication. For more information, please contact onepress@plos.org.

With kind regards,

Sabrina Sarrocco

Academic Editor

PLOS ONE

Additional Editor Comments (optional):

Reviewers' comments:

# Editor report: Acceptance letter

**DOI:** 10.1371/journal.pone.0230801.r004

**Authors:** Sabrina Sarrocco

27 Mar 2020

PONE-D-19-20356R1 

 Effect of physio-chemical seed treatments on opium poppy downy mildews caused by *Peronospora meconopsidis* and *P. somniferi.*

Dear Dr. Thangavel:

I am pleased to inform you that your manuscript has been deemed suitable for publication in PLOS ONE. Congratulations! Your manuscript is now with our production department. 

If your institution or institutions have a press office, please notify them about your upcoming paper at this point, to enable them to help maximize its impact. If they will be preparing press materials for this manuscript, please inform our press team within the next 48 hours. Your manuscript will remain under strict press embargo until 2 pm Eastern Time on the date of publication. For more information please contact onepress@plos.org.

For any other questions or concerns, please email plosone@plos.org. 

Thank you for submitting your work to PLOS ONE.

With kind regards,

PLOS ONE Editorial Office Staff

on behalf of

Dr Sabrina Sarrocco 

Academic Editor

PLOS ONE
